# Publisher Correction: Crosstalk between salicylic acid signalling and the circadian clock promotes an effective immune response in plants

**DOI:** 10.1038/s44323-025-00026-4

**Published:** 2025-03-07

**Authors:** Olivia J. P. Fraser, Samantha J. Cargill, Steven H. Spoel, Gerben van Ooijen

**Affiliations:** The University of Edinburgh, School of Biological Sciences, Max Born Crescent, EH9 3BF Edinburgh, UK

**Keywords:** Plant sciences, Diseases

Correction to: *npj Biological Timing and Sleep* 10.1038/s44323-024-00006-0, published online 02 September 2024

The Data Availability statement was missing and should have read, “Data sharing is not applicable to this article as no datasets were generated or analysed during the current study. Raw luminescence data can be obtained from the corresponding author upon reasonable request.”. The original article has been updated. The publisher apologises for the inconvenience caused to our authors and readers.

